# Retrospective longitudinal and comparative observational study between gastric bypass surgery and sleeve gastrectomy: 5-year post-operative follow-up

**DOI:** 10.1590/0102-67202025000016e1885

**Published:** 2025-06-27

**Authors:** Admar CONCON, Sergio Henrique Bastos DAMOUS, Jose Pinhata OTOCH, Matheus Borges CORONADO, Idiberto José ZOTARELLI, Manoel Passos GALVÃO, Vítor Ottoboni BRUNALDI, Everson Luiz Almeida ARTIFON

**Affiliations:** 1 Hospital e Maternidade Galileo, Surgery Unit – Valinhos (SP), Brazil.; 2 Universidade de São Paulo, Faculty of Medicine, Department of Surgery – São Paulo (SP), Brazil.; 3 Universidade Nove de Julho – São Paulo (SP), Brazil.; 4 Universidade Estadual Paulista, Institute of Biosciences, Letters and Exact Sciences – São José do Rio Preto (SP), Brazil;; 5 Orlando Health Hospital, Bariatric Surgery – Orlando (FL), USA.

**Keywords:** Gastric Bypass, Obesity, Bariatric Surgery, Esophagitis, Peptic, Barrett's Esophagus, Derivação Gástrica, Obesidade, Cirurgia Bariátrica, Esofagite Péptica, Esôfago de Barrett

## Abstract

**Background:**

Among the 2.0 billion overweight individuals in the world, Brazil ranks fifth in the number of obese people, therefore requiring treatment options for obesity.

**Aim:**

The aim of this study was to compare the percentage of total body weight loss (%TWL), change in body mass index (BMI), percentage of excess weight loss (%EWL), incidence of reflux esophagitis, and occurrence of Barrett's esophagus in obese patients undergoing gastric bypass (Roux-en-Y gastric bypass [RYGB]) and sleeve gastrectomy (SG), both techniques by videolaparoscopy.

**Methods:**

The study included 100 consecutive patients who underwent RYGB and SG techniques, totaling 200 patients, and were followed up for 60 months, from June 2013 to July 2018.

**Results:**

The frequency of gastroesophageal reflux disease (GERD) was lower in RYGB patients (p<0.05). At 60 months, the %EWL was 77.4±13.3 kg (RYGB) versus 80.5±17.5 kg (SG) (p<0.05). The BMI data were statistically significantly different between groups after 5 years (28.5±3.9 kg/m^2^ in RYGB and 31.9±5.3 kg/m^2^ in SG groups, p<0.05). During the follow-up, the RYGB showed higher %EWL compared to the SG (at 60 months, 80.1% vs. 59.1%, respectively, p<0.05). The %TWL was 30% for the RYGB and 19.7% for the SG (p<0.05). The RYGB had a lower frequency of reflux esophagitis and Barrett's esophagus.

**Conclusions:**

The RYGB technique showed greater absolute weight loss, %TWL, BMI reduction, and %EWL in higher obesity classes compared to the SG technique. Additionally, RYGB had a lower frequency of reflux esophagitis and Barrett's esophagus.

## ARTICLE HIGHLIGHTS

- The Roux-en-Y gastric bypass technique showed higher results compared to the sleeve gastrectomy technique.
- The Roux-en-Y gastric bypass technique showed lower frequency of reflux esophagitis and Barrett's esophagus than sleeve gastrectomy.
- The sleeve gastrectomy should indeed be considered a surgical alternative for the treatment of obesity, taking care when selecting patients to minimize post-surgical GERD.

## CENTRAL MESSAGE

Currently, 55% of the Brazilian population is above the body mass index (BMI)-25 kg/m^2^ threshold, and as of 2030, most Americans are expected to be obese. Less than 2% of the patients with clinical indications to undergo bariatric surgery receives treatment. Due to its relative technical simplicity, reproducibility, and adequate weight loss outcomes, laparoscopic sleeve gastrectomy (LSG) has rapidly become the dominant bariatric surgery worldwide. The Roux-en-Y gastric bypass (RYGB) is the most ancient bariatric surgery still being performed. It promotes sustainable weight loss and reliable improvements in metabolic and cardiovascular diseases.

## PERSPECTIVES

The Roux-en-Y gastric bypass (RYGB) promotes better weight loss than laparoscopic sleeve gastrectomy (LSG) throughout a 5-year follow-up. Lower levels of total cholesterol, low-density lipoprotein (LDL), triglycerides, serum iron, fasting glucose, and insulin accompany the increased weight loss. LSG carries higher rates of proton pump inhibitor (PPI) use and heartburn than RYGB.

## VISUAL ABSTRACT

**Figure:**
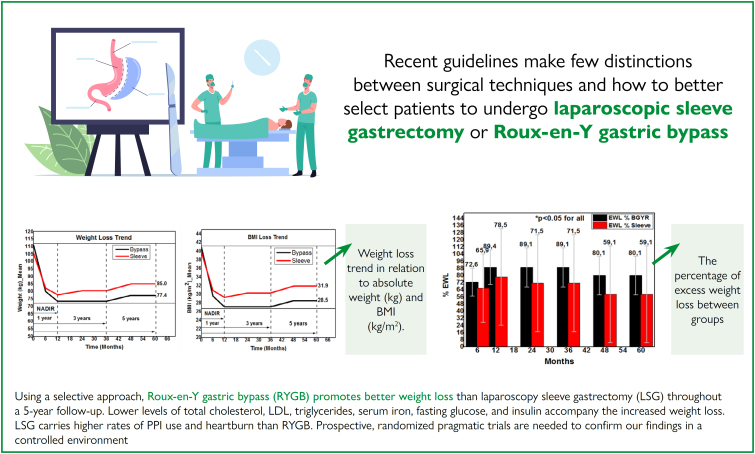

## RESUMO

**Racional:**

Dentre os 2,0 bilhões de indivíduos com sobrepeso no mundo, O Brasil ocupa o quinto lugar em número de obesos, portanto, necessitando de opções de tratamento para obesidade.

**Objetivos:**

Comparar a porcentagem de perda de peso corporal total, alteração do índice de massa corporal (IMC), a porcentagem de perda de excesso de peso (%PEP), incidência de esofagite de refluxo e a ocorrência esôfago de Barrett em obesos submetidos às técnicas de bypass gástrico (RYGB) e a gastrectomia vertical (GV) por videolaparoscopia.

**Métodos:**

Foram incluídos no estudo 100 pacientes consecutivos submetidos às técnicas RYGB e o LSG, totalizando 200 pacientes, seguidos durante 60 meses, de junho de 2013 a julho de 2018.

**Resultados:**

A frequência de doença do refluxo gastroesofágico (DRGE) registrada foi menor em pacientes submetidos à RYGB (p < 0,05). Aos 60 meses, a perda absoluta de peso foi de 77,4±13,3 kg (RYGB) versus 80,5±17,5 (GV) (p<0,05). Os dados do IMC foram, após 5 anos, 28,5 ± 3,9 kg/m2 no RYGB e 31,9 ± 5,3 kg/m2 nos grupos GV, p<0,05. A técnica RYGB mostrou maiores %PEP em comparação à técnica GV (aos 60 meses, 80,1% vs. 59,1%, respectivamente, p<0,05). A porcentagem de perda do peso total (%PEP) foi de 30% para à técnica RYGB e 19,7% para a GV (p<0,05). A técnica RYGB teve menor frequência de esofagite de refluxo e esôfago de Barrett.

**Conclusões:**

A técnica RYGB mostrou maior perda de peso absoluta, porcentagem de perda de peso corporal total, redução do IMC e %PEP em comparação à técnica GV; além de menor frequência de esofagite de refluxo e esôfago de Barrett.

## INTRODUCTION

Despite all medical efforts, the obesity pandemic outweighs the health system's capacity to respond. Currently, 55% of the Brazilian population is above the body mass index (BMI)-25 kg/m^2^ threshold, and as of 2030, most Americans are expected to be obese^9^. Less than 2% of the patients with clinical indications to undergo bariatric surgery receives treatment^3^. These alarming numbers have triggered the development and refinement of techniques to address moderate and severe obesity^21^. Laparoscopic sleeve gastrectomy (LSG) is one of them. Although it has been used for over three decades as the first step of the duodenal switch procedure, the LSG has become a definite procedure since the early 2000s^11^. Due to its relative technical simplicity, reproducibility, and adequate weight loss outcomes, LSG has rapidly become the dominant bariatric surgery worldwide^2,3^.

Roux-en-Y gastric bypass (RYGB) is the most ancient bariatric surgery still being performed. It promotes sustainable weight loss and reliable improvements in metabolic and cardiovascular diseases^5^. Moreover, since this technique bypasses the duodenum and part of the proximal jejunum, it is also considered a metabolic procedure^14^.

Recent guidelines make few distinctions between surgical techniques and how to better select patients to undergo LSG or RYGB^10,13^. Randomized trials comparing LSG and RYGB have shown slightly better weight loss and cardiovascular improvement in favor of the latter. Also, LSG conveys an increased risk of *de novo* or worsening gastroesophageal reflux disease (GERD)^19,20^. Still, random allocation does not simulate the real-world scenario and usually attenuates the differences in the measures of effect^8^. Forcefully allocating patients to receive one therapy or the other does not allow physicians to extract the most of each procedure. Therefore, we conducted a retrospective cohort study from a single-center bariatric center to compare real-world data between LSG and RYGB.

## METHODS

### Study design and ethics

This is a single-center (Hospital e Maternidade Galileo – Valinhos (SP), Brazil), retrospective cohort study comparing surgical and clinical outcomes up to 5 years of consecutive patients undergoing RYGB or LSG. The study was approved by the Ethics Committee of the University of Sao Paulo (number 5-538-666) and was conducted according to the principles of the Declaration of Helsinki.

### Eligibility

A total of 100 LSG and 100 RYGB consecutive patients undergoing treatment from June 2013 to July 2018 were included. We separated the last 100 bypasses operated on in December/2014 and the first 100 sleeves operated on in January/2014. Adults between 18 and 65 years old with obesity class II or III (BMI>35 kg/m^2^) and surgically fit were included. All patients signed the informed consent form to undergo bariatric surgery.

### Multidisciplinary team and follow-up

The multidisciplinary team included bariatric surgeons, endocrinologists, psychologists, nutritionists, and nurses. The follow-up was standardized despite the type of bariatric procedure and included in-person follow-up at 1, 2, and 4 weeks and 3, 6, 9, 12, 18, and 24 months. Then, patients were instructed to return yearly for follow-up with the bariatric surgeon and endocrinologist. All patients received perennial oral supplements per previous guidelines^18^.

### Surgical techniques

Both LSG and RYGB were carried out with standardized laparoscopic surgical techniques. For RYGB, patients were placed on the French position and surgery was performed with five trocars^16^. Anastomosis was performed with an Endo GIA stapler, 2 cm long, with the closure of the stapler hole with polydioxanone (PDS) 2.0 thread, sutured in two planes (total plane and then seromuscular). Endostaplers were employed to section the stomach and create the anastomoses. The alimentary limb measured 150 cm and the biliopancreatic limb 100 cm^1^. For the LSG, we adopted the same number of trocar and patients’ position. The gastric sleeve was described as the distance from the pylorus and the first staple (4 cm). We employed a 36 French Fouchet bougie to calibrate the stapling line, which was later oversewn with 3-0 PDS sutures. No abdominal drains were placed. One experienced surgeon carried out all the procedures.

### Clinical outcomes and definitions

The primary endpoint was weight loss at 5 years according to percentage of total weight loss (%TWL), percentage of excess weight loss (%EWL) considering the weight at BMI-25 kg/m^2^, absolute weight loss (AWL) (kg), and BMI reduction (kg/m^2^). Secondary outcomes included the rate of erosive esophagitis and Barrett's esophagus, improvement in weight-related comorbidities, and adverse events (AEs). AEs were classified as immediate (<24 h), early (>24 h, <30 days), and late (>30 days). The severity of AEs was also determined based on previously validated scales^7^.

### Statistical analysis

An experienced statistician conducted all statistical analyses. We built a spreadsheet for data extraction, which was later exported to the software Minitab 18^®^ (version 18, Minitab, LLC, State College, Pennsylvania, USA) and OriginPro^®^ 9 (DPR Group, Inc., Northampton, MA, USA). Initially, we performed a descriptive statistical analysis of the data (mean, standard deviation, median, maximum and minimum values, and percentage) and applied the Anderson-Darling normality test for all numerical variables. Then, Kruskal-Wallis or equivalent parametric tests were applied accordingly with Bonferroni corrections as needed. A p<0.05 was considered statistically significant for a 95% confidence interval (CI).

## RESULTS

A total of 200 patients (100 LSG and 100 RYGB) with a mean age and BMI of 38.1±9.8 years and 40.3±4.7 kg/m^2^, respectively, were included in the study. RYGB patients were slightly younger (36.3±9.2 vs. 39.9±10.1 years, p=0.01), were heavier (baseline weight 111.1±16.2 vs. 105.6±19.3 kg, p=0.03), had more excess weight (43.1±12.7 vs. 39.3±14.8 kg, p=0.05), and had higher prevalence of heartburn symptoms (43% vs. 12%, p<0.001) and PPI use (21% vs. 7%, p=0.004). Table 1 summarizes the patients’ preoperative data.

**Table 1:** Demographic table of patients included in the study.

Variables		Group		Total (n=200)	p-value
		RYGB (n=100)	LSG (n=100)		
Age (years)		36.3±9.2	39.9±10.1	38.1±9.8	**0.01**
Gender (F/M), n		84/16	87/13	171/29	0.54
Weight (kg)		111.1±16.2	105.6±19.3	108.3±18	**0.03**
BMI (kg/m²)		40.9±4.6	39.7±4.7	40.3±4.7	0.06
Excess weight (kg)		43.1±12.7	39.3±14.8	41.2±13.9	**0.05**
Comorbidities (n)					
	Hypothyroidism	9	11	20	0.63
	Steatosis by US	67	65	132	0.45
	Hypertension	38	47	85	0.19
	Diabetes mellitus	16	14	33	0.34
	Metabolic syndrome	30	30	60	>0.99
	Sleep apnea	58	48	106	0.15
	Heartburn	43	12	55	**<0.001**
LA A esophagitis (n)		32	30	62	0.76
LA B esophagitis (n)		1	4	5	0.36
LA C esophagitis (n)		2	3	5	>0.99
Hiatal hernia (n)		2	1	3	>0.99
PPI use (n)		21	7	28	**0.004**
Total cholesterol		187.5±40.8	198.7±36.5	193.1±39	**0.04**
LDL		110.7±31.9	114.5±34.9	112.5±33.3	0.45
HDL		49.3±11	49.6±11.7	49.5±11.3	0.87
Triglycerides		141.2±75	157.2±72.1	149.1±73.9	0.12
Fasting glucose (mg/dL)		102.8±61.8	98.9±29.2	100.8±48.4	0.57
Fasting insulin		21.9±21.3	20.8±24.3	21.4±22.8	0.73
HbA1c (%)		7.18±2.12	6.5±1.82	6.95±1.94	0.65

RYGB: Roux-en-Y gastric bypass; LSG: Laparoscopic sleeve gastrectomy; LA: Los Angeles Classification; LDL: low-density lipoprotein; HDL: high-density lipoprotein; PPI: proton pump inhibitor; BMI: body mass index; US: ultrasound. Statistically significant values are denoted in bold.

**Figure 1:**
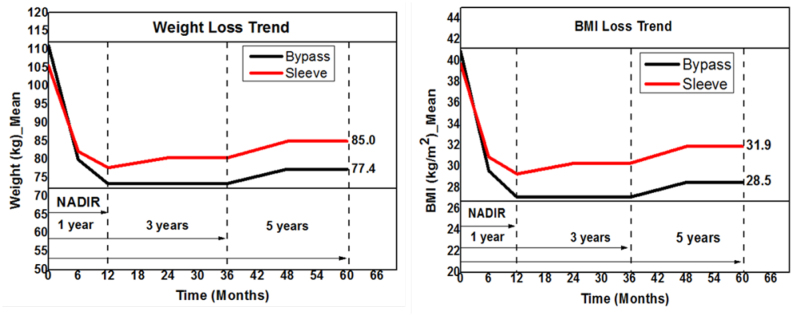
Weight loss trend in relation to absolute weight (kg) and BMI (kg/m^2^).

**Figure 2:**
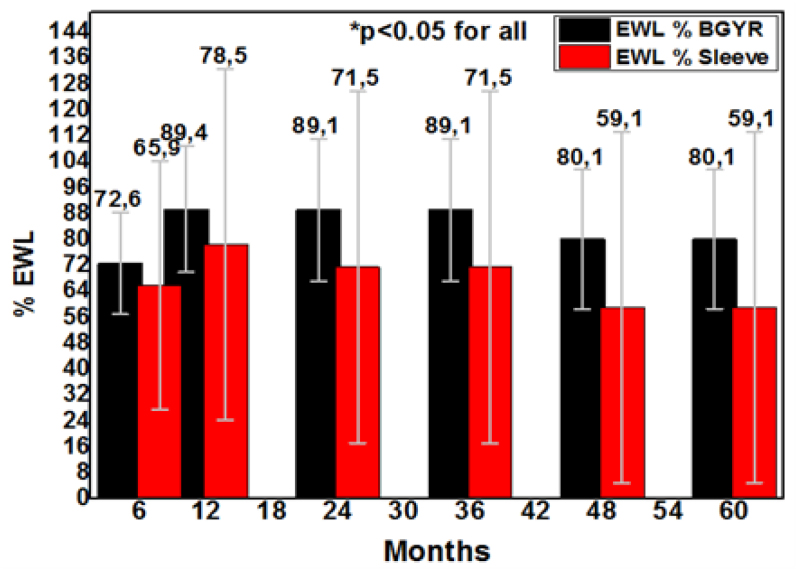
The percentage of excess weight loss between groups.

Throughout the 5-year follow-up period, RYGB patients lost significantly more weight in terms of absolute weight, BMI, %TWL, and %EWL (Table 2). When analyzing trends, the difference in weight loss started between 6 and 12 months, progressed between 12 and 24 months, and remained constant afterward (Figures 1 and 2).

**Table 2:** Summary of weight loss outcomes and comparisons between Roux-en-Y gastric bypass and Laparoscopic sleeve gastrectomy.

Variables		Group	
		RYGB	LSG
6 months (n)		99	100
	Weight	80±9.9	82.2±17.9
	BMI	29.6±2.9	30.9±4.5
	%EWL	72.6±15.7	65.9±38.3
	%TWL	27.3±5.8	22.4±6
12 months (n)		100	99
	Weight	73.4±10.5	77.8±16
	BMI	27.1±3	29.3±4.3
	%EWL	89.4±19.5	78.5±54.2
	%TWL	33.6±6.6	26.4±7.6
24–36 months (n)		100	98
	Weight	73.5±12.1	80.5±17.5
	BMI	27.1±3.4	30.3±4.8
	%EWL	89.1±22	71.5±54.3
	%TWL	33.5±7.9	23.9±8.8
48–60 months (n)		98	100
	Weight	77.4±13.3	85±18.9
	BMI	28.5±3.9	31.9±5.3
	%EWL	80.1±21.5	59.1±54.2
	%TWL	30±8	19.7±10.1
p-values (between groups)			
	Weight	0.008	
	BMI	<0.001	
	%EWL	0.008	
	%TWL	<0.001	
Post-hoc analysis (Bonferroni correction)		Time points withp<0.05 between groups	
	Weight	24–36 mo and 48–60 mo	
	BMI	12 mo, 24–36 mo, and 48–60 mo	
	%EWL	24–36 mo and 48–60 mo	
	%TWL	All time points	

RYGB: *Roux-en-Y gastric bypass; LSG: Laparoscopic sleeve gastrectomy; BMI*: *body mass index; %EWL: % excess weight loss; %TWL*: *%total weight loss;* Mo: months.

Within 12 months, AWL at nadir was 73.4±10.5 kg and 77.8±16 kg for the RYGB and LSG groups, respectively. At 60 months, the AWL was 77.4±13.3 kg (RYGB) versus 80.5±17.5 kg (LSG) (p<0.05). Accordingly, BMI data were statistically significantly different between groups after 5 years (28.5±3.9 kg/m^2^ in RYGB and 31.9±5.3 kg/m^2^ in LSG groups, p<0.05). At all time points throughout follow-up, RYGB showed higher %EWL compared to LSG (at 60 months, 80.1% vs. 59.1%, respectively, p<0.05). The %TWL was 30% for the RYGB and 19.7% for the LSG (p<0.05) (Table 2).

Throughout 5 years of follow-up, RYGB patients presented significantly lower levels of total cholesterol, low-density lipoprotein (LDL), triglycerides, serum iron, fasting glucose, and insulin. The rates of GERD and heartburn symptoms and proton pump inhibitor (PPI) use were also significantly lower compared to LSG patients. However, the rates of erosive esophagitis did not differ between groups. As to side effects, eventual vomiting was more frequent in RYGB individuals (Table 3).

**Table 3:** Laboratory and clinical data from 6 to 60 months, comparing Roux-en-Y gastric bypass and Laparoscopic sleeve gastrectomy.

Variables		Follow-up				p-value	Time points with p<0.05 (post-hoc)
		6 months	12 months	24–36 months	48–60 months		
Total cholesterol							
	RYGB (n)	155.5±31 (100)	157.1±31.1 (100)	163.8±28.1 (97)	174.7±35.3 (95)	**<0.001**	6 mo, 12 mo, and 24–36 mo
	LSG (n)	189.3±32.6 (100)	193.1±33 (98)	189±31.3 (98)	168.3±49 (91)		
LDL							
	RYGB (n)	89.5±27.1 (94)	84.7±23.7 (95)	86±24.2 (89)	91.8±30.7 (88)	**<0.001**	6 mo and 12 mo
	LSG (n)	115.2±33.6 (85)	117.9±31.2 (81)	107.2±28.4 (91)	94.9±39.9 (93)		
HDL							
	RYGB (n)	50±15.2 (99)	55.1±13.6 (98)	61.8±12.7 (95)	66±17.3 (94)	0.29	
	LSG (n)	48.9±10.6 (98)	55.1±11.6 (94)	60.8±19.6 (95)	75.8±45.4 (93)		
Triglycerides							
	RYGB (n)	89.6±40.8 (98)	82.9±36.9 (99)	78.6±35.5 (97)	87.5±76.2 (95)	**<0.001**	All time points
	LSG (n)	107.7±46.6 (100)	98.9±45.7 (96)	98.8±50.3 (95)	103.9±53.1 (55)		
Serum iron							
	RYGB (n)	122.1±130.2 (95)	107.2±107.3 (97)	89.6±156.4 (94)	52.4±65.7 (94)	**0.001**	48–60 mo
	LSG (n)	121±98.1 (85)	126.4±124.6 (75)	93±99.9 (77)	259±335 (73)		
Vitamin B12							
	RYGB (n)	520.9±648.3 (98)	606.9±609.7 (98)	643.4±625.3 (97)	564.9±491.3 (95)	0.09	
	LSG (n)	557.7±612.7 (95)	481.3±452.2 (90)	588.6±422.1 (89)	396.3±491.8 (85)		
Fasting glucose							
	RYGB (n)	84±13.7 (99)	81.8±10.5 (99)	82.6±9.3 (97)	84.9±9.4 (95)	**0.01**	All time points
	LSG (n)	85.4±14.5 (99)	92.4±65.6 (96)	94±77.4 (92)	95.2±86.9 (89)		
Fasting insulin							
	RYGB (n)	8.1±10.9 (97)	6.4±7.9 (96)	7.3±9.7 (92)	8.2±11.4 (87)	**<0.001**	All time points
	LSG (n)	17±47.1 (93)	27.8±81.7 (72)	14.5±26.8 (85)	11.7±18.9 (51)		
Vitamin D							
	RYGB (n)	55.4±100.2 (92)	37.1±32.8 (84)	30.4±11.2 (84)	26.6±8.6 (85)	**0.01**	All time points
	LSG (n)	38.6±17.6 (49)	23.2±13.7 (62)	28.6±8.2 (74)	28.5±51.9 (79)		
Folic acid							
	RYGB (n)	27.5±92.7 (99)	15.7±12.1 (98)	22.6±73.9 (92)	15.3±9.2 (90)	0.14	
	LSG (n)	18.3±59.1 (91)	36.3±35.8 (92)	14±10.1 (88)	37±38.6 (69)		
Zinc							
	RYGB (n)	105.6±120.5 (98)	82.7±13.5 (94)	90.2±78.7 (90)	81.4±12.3 (87)	0.27	
	LSG (n)	94.8±77.1 (89)	81.8±20.6 (63)	86.6±15.3 (79)	77.9±31.3 (50)		
AST							
	RYGB (n)	20.7±8.5 (30)	23±8.7 (24)	23.4±10.2 (27)	30.6±33.3 (20)	0.06	
	LSG (n)	17.5±4.5 (19)	18.3±9 (16)	16.2±5.6 (17)	28.4±21.5 (25)		
ALT							
	RYGB (n)	23.5±10.6 (37)	23±12.1 (29)	22.4±11.4 (30)	25.7±19.7 (30)	0.30	
	LSG (n)	21.3±15.2 (22)	22.9±10.4 (14)	18±7.7 (18)	23.2±17 (26)		
Gamma GT							
	RYGB (n)	21.6±22.8 (28)	21.9±16.9 (21)	18.8 ±7.7 (21)	26.3±29.8 (13)	0.17	
	LSG (n)	19.9±8.2 (18)	41.6±31.6 (11)	30.7±29.9 (12)	24.9±17.4 (11)		
Hypertension							
	RYGB (n)	0 (96)	0 (99)	0 (98)	2 (99)	0.69	
	LSG (n)	0 (99)	2 (99)	2 (99)	0 (98)		
Diabetes mellitus							
	RYGB (n)	2 (98)	1 (99)	2 (98)	3 (99)	0.88	
	LSG (n)	2 (98)	1 (99)	2 (99)	2 (98)		
Metabolic syndrome							
	RYGB (n)	4 (96)	1 (99)	1 (99)	3 (96)	0.07	
	LSG (n)	5 (98)	5 (96)	7 (99)	9 (99)		
GERD/heartburn							
	RYGB (n)	8 (100)	5 (100)	14 (100)	17 (98)	<0.001	All time points
	LSG (n)	17 (100)	21 (99)	29 (98)	29 (99)		
LA A esophagitis							
	RYGB (n)	9 (100)	3 (100)	7 (100)	9 (98)	0.51	
	LSG (n)	8 (100)	3 (98)	16 (98)	9 (98)		
PPI use							
	RYGB (n)	28 (100)	28 (100)	11 (100)	9 (98)	**<0.001**	6 mo
	LSG (n)	72 (100)	46 (97))	18 (98)	17 (98)		
Epigastric pain							
	RYGB (n)	18 (100)	17 (100)	19 (100)	16 (98)	0.09	
	LSG (n)	15 (100)	14 (99)	8 (98)	14 (100)		
Vomiting							
	RYGB (n)	20 (100)	13 (100)	18 (100)	12 (97)	**0.01**	All time points
	LSG (n)	9 (100)	6 (99)	7 (98)	10 (100)		
Gastritis							
	RYGB (n)	10 (100)	9 (99)	2 (100)	3 (98)	0.27	
	LSG (n)	12 (100)	5 (99)	5 (98)	9 (99)		

LDL: low-density lipoprotein; HDL: high-density lipoprotein; AST: aspartate aminotransferase; ALT:alanine aminotransferase; RYGB: Roux-en-Y gastric bypass; LSG: laparoscopic sleeve gastrectomy; LA: Los Angeles Classification; PPI: proton pump inhibitor; GERD: gastroesophageal reflux disease. Statistically significant values are denoted in bold.

### Adverse events

AEs were classified as immediate (up to 7 days), early (7–30 days), and late (after 30 days). During the follow-up period, we had five cases of reoperation in the bypass group (5%), three cases of subocclusion, two due to adhesions and one due to hernia in the mesentery, one case of hematoma in the pouch staple line, and one case of incisional hernia in the trocar. In patients undergoing LSG, we had one case that required revision surgery to transform the RYGB, due to weight regain.

## DISCUSSION

Although the clinical comparison between LSG and RYGB is not new, non-academic, real-world, long-term data are still scarce. Moreover, few studies have evaluated outcomes in such a comprehensive manner, including not only clinical but also laboratory, clinical, and endoscopic. Finally, our loss to follow-up was minimal in both groups. Altogether, our study was able to provide reliable, practical data to help surgeons better decide between surgical techniques.

First, our demographic table brings important information regarding the selection criteria we utilized. More severe obesity, younger age, presence of GERD-related symptoms, and PPI use favored patients to receive RYGB rather than LSG. Although there was no baseline difference in terms of metabolic disease and hypertension, these are two additional factors that favor RYGB, as previous data show higher resolution rates with this technique^6,20^.

One of the advantages of LSG over RYGB is the absence of intestinal bypass, which renders this procedure bariatric but non-malabsorptive. As a result, the rates of nutritional deficiencies are markedly lower, which may exempt those patients from perennial oral or parenteral supplementation^18^. Our results reinforce that, as we demonstrated lower serum iron levels, although no iron deficiency cases were reported.

Noteworthy, RYGB patients were supplemented, indicating that:

1. Supplementation is necessary but still not enough to counterbalance malabsorption and
2. Long-term laboratory surveillance should be implemented.

LSG has been consistently associated with worsening or postoperative *de novo* GERD^4^. Some authors advocate that selective indication of LSG could mitigate this AE^12^. However, in our study, LSG patients had higher rates of heartburn and PPI use during follow-up despite a highly selective indication and higher baseline rates of heartburn and PPI use in the RYGB group. This finding adds to previous literature showing that LSG is, per se, a refluxogenic procedure^15,17^.

As to weight loss, RYGB consistently showed better outcomes throughout follow-up. Despite a significantly higher baseline weight and borderline higher baseline BMI, those patients presented significantly lower weight and BMI at 5 years. When analyzing weight trends (Figure 1), it is notable that the difference between groups occurs within the first 2 years. After that, the weight trends parallel with RYGB on the lower end. This finding is in accordance with previous literature^5^ and suggests that this time point could be used to assess clinical success and to indicate revisional or conversion procedures in the case of inadequate weight loss.

Our study is not exempt from limitations. First, as a retrospective study, selection bias played an important role. Although one could argue against generalizability, we advocate that it comes to our advantage as the selective indication is the current practice in real-world and non-academic centers. Second, our sample size was not large enough to detect minor differences such as rates of metabolic syndrome. Still, we had enough power to detect differences in our primary endpoints, our first and foremost goal.

## CONCLUSIONS

Using a selective approach, RYGB promotes better weight loss than LSG throughout a 5-year follow-up. Lower levels of total cholesterol, LDL, triglycerides, serum iron, fasting glucose, and insulin accompany the increased weight loss. LSG carries higher rates of PPI use and heartburn than RYGB. Prospective, randomized pragmatic trials are needed to confirm our findings in a controlled environment.

## Data Availability

The datasets generated and/or analyzed during the current study are available from the corresponding author upon reasonable request.
